# Tranexamic Acid in Civilian Trauma Care in the California Prehospital Antifibrinolytic Therapy Study

**DOI:** 10.5811/westjem.2018.8.39336

**Published:** 2018-09-10

**Authors:** Michael M. Neeki, Fanglong Dong, Jake Toy, Reza Vaezazizi, Joe Powell, David Wong, Michael Mousselli, Massoud Rabiei, Alex Jabourian, Nichole Niknafs, Michelle Burgett-Moreno, Richard Vara, Shanna Kissel, Xian Luo-Owen, Karen R. O’Bosky, Daniel Ludi, Karl Sporer, Troy Pennington, Tommy Lee, Rodney Borger, Eugene Kwong

**Affiliations:** * Arrowhead Regional Medical Center, Department of Emergency Medicine, Colton, California; † Arrowhead Regional Medical Center, Department of Surgery, Colton, California; ‡ California University of Sciences and Medicine, Colton, California; § Inland Counties Emergency Medical Agency, San Bernardino, California; ¶ Riverside County Emergency Services Agency, Riverside, California; || City of Rialto Fire Department, Rialto, California; # Loma Linda University Medical Center, Department of General Surgery, Loma Linda, California; ** Riverside University Health System Medical Center, Department of Surgery, Moreno Valley, California; †† Alameda County Emergency Medical Services Agency, San Leandro, California

## Abstract

**Introduction:**

Hemorrhage is one of the leading causes of death in trauma victims. Historically, paramedics have not had access to medications that specifically target the reversal of trauma-induced coagulopathies. The California Prehospital Antifibrinolytic Therapy (Cal-PAT) study seeks to evaluate the safety and efficacy of tranexamic acid (TXA) use in the civilian prehospital setting in cases of traumatic hemorrhagic shock.

**Methods:**

The Cal-PAT study is a multi-centered, prospective, observational cohort study with a retrospective comparison. From March 2015 to July 2017, patients ≥ 18 years-old who sustained blunt or penetrating trauma with signs of hemorrhagic shock identified by first responders in the prehospital setting were considered for TXA treatment. A control group was formed of patients seen in the five years prior to data collection cessation (June 2012 to July 2017) at each receiving center who were not administered TXA. Control group patients were selected through propensity score matching based on gender, age, Injury Severity Scores, and mechanism of injury. The primary outcome assessed was mortality recorded at 24 hours, 48 hours, and 28 days. Additional variables assessed included total blood products transfused, the hospital and intensive care unit length of stay, systolic blood pressure taken prior to TXA administration, Glasgow Coma Score observed prior to TXA administration, and the incidence of known adverse events associated with TXA administration.

**Results:**

We included 724 patients in the final analysis, with 362 patients in the TXA group and 362 in the control group. Reduced mortality was noted at 28 days in the TXA group in comparison to the control group (3.6% vs. 8.3% for TXA and control, respectively, odds ratio [OR]=0.41 with 95% confidence interval [CI] [0.21 to 0.8]). This mortality difference was greatest in severely injured patients with ISS >15 (6% vs 14.5% for TXA and control, respectively, OR=0.37 with 95% CI [0.17 to 0.8]). Furthermore, a significant reduction in total blood product transfused was observed after TXA administration in the total cohort as well as in severely injured patients. No significant increase in known adverse events following TXA administration were observed.

**Conclusion:**

Findings from the Cal-PAT study suggest that TXA use in the civilian prehospital setting may safely improve survival outcomes in patients who have sustained traumatic injury with signs of hemorrhagic shock.

## INTRODUCTION

In the United States (U.S.), traumatic injury is the leading cause of death and disability among those aged 1 to 44 years old.^1^ Among trauma victims, hemorrhage accounts for 30% to 40% of the mortality.^2^–^4^ Within the prehospital setting, hemorrhage is one of the top causes of death and comprises the largest portion of preventable deaths.^2^,^3^ Significant blood volume loss leads to the depletion of coagulation factors and dysregulation of the coagulation system. Combined, these factors threaten the body’s ability to maintain hemodynamic stability and may result in cardiovascular collapse.

The burden of trauma-induced coagulopathies (TIC) has been demonstrated in more than half of trauma patients following arrival to trauma centers and has been associated with a significant increase in the risk of trauma-induced mortality.^5^–^9^ Historically, paramedics have not had access to medications that specifically target the reversal of TIC.^3^,^4^ As biotechnological advances enable better detection and understanding of TIC, a group of patients has been identified that may benefit from early reversal of traumatic coagulopathies, leading to a possible reduction in associated mortality.^8^,^10^–^12^

Tranexamic acid (TXA) is a synthetic derivative that inhibits fibrinolysis and has been shown to be effective in the hospital setting in the treatment of hemorrhagic shock. In 2010 the Clinical Randomization of an Antifibrinolytic in Significant Hemorrhage-2 (CRASH-2) trial suggested that TXA was associated with a 1.5% reduction (14.5% vs. 16%) in all-cause mortality at 28 days when administered within eight hours of injury without an increase in thromboembolic events.^13^ In 2011 a post-hoc analysis showed that early TXA treatment within three hours from the time of injury in the hospital setting resulted in a 1.6% decrease in death due to bleeding; the reduction in mortality increased to 2.4% if administered within one hour from injury.^14^

Despite evidence surrounding hospital TXA use, a gap in knowledge exists surrounding the prehospital TXA use in the civilian setting. Multiple small studies have demonstrated the feasibility of prehospital TXA administration including the ability of paramedics to identify candidates with signs of hemorrhagic shock.^15^–^18^ Two recent investigations focusing on civilian injuries in Germany and Japan further suggest that prehospital TXA use may reduce mortality in severely injured trauma victims.^19^–^20^ However, their retrospective nature and the lack of standardized dosages and algorithms for TXA administration limited the generalizability of those studies. This paucity of out-of-hospital data has limited the widespread implementation of TXA into U.S. civilian prehospital-care protocols.

The California Prehospital Antifibrinolytic Therapy (Cal-PAT) study was designed to evaluate the safety and efficacy of TXA use in the civilian prehospital setting in traumatic hemorrhagic shock. A preliminary report during ongoing data collection from the Cal-PAT study was published in 2017.^21^ This current study reports the final findings of the prehospital component of the Cal-PAT study. We hypothesized that the prehospital administration of TXA in cases of traumatic hemorrhagic shock would be associated with a decrease in mortality.

**Population Health Research Capsule**

What do we already know about this issue?

Prior studies assessing tranexamic acid (TXA) use in civilian and military trauma resuscitation demonstrate a promising effect on mortality reduction and a limited side-effect profile.

What was the research question?

What is the impact and feasibility of prehospital TXA use in trauma-induced hemorrhagic shock within North American emergency medical services standards?

What was the major finding of the study?

TXA use was associated with improved survival in traumatic hemorrhagic shock and a decrease in blood product utilization.

How does this improve population health?

Traumatic injury is a major cause of death in both developed and developing nations. TXA use represents a feasible measure toward reducing loss of life due to traumatic exsanguinating injury.

## METHODS

### Cal-PAT Study Overview

The Cal-PAT study was a multi-centered, prospective, observational cohort study with a retrospective comparison. The study was initiated in March 2015 in two Southern California counties–San Bernardino and Riverside. In early 2016 Alameda County joined the study. All eight receiving centers are designated Level I and Level II trauma centers. A total of 30 emergency medical services (EMS) agencies were involved across all counties. Current data collection for this study concluded in July 2017 in all counties. Within the prehospital setting, the California Emergency Medical Services Authority approved TXA to be included in EMS protocols as a standard treatment for all trauma patients showing signs of hemorrhagic shock. TXA administration was carried out uniformly among all participating EMS agencies. The institutional review board at each trauma center approved CAL-PAT study protocols, including the incorporation of TXA into the massive transfusion protocol at each center as a standard of care for trauma patients and allowed for research data collection with a waiver of consent.

### Data collection, Protocols, Outcomes

**Figure 1:**
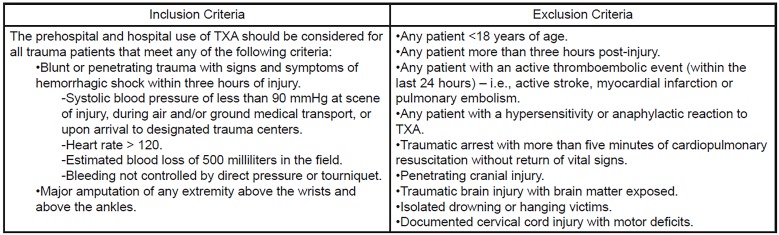
Inclusion and exclusion criteria provided to first responders in the field and clinicians at receiving trauma centers. *TXA*, tranexamic acid.

All patients ≥18 years old who sustained blunt or penetrating trauma with signs of hemorrhagic shock were considered for TXA treatment upon meeting enrollment criteria (Figure 1). Patient selection in the prehospital setting was determined by paramedics on ambulances or by registered nurses on helicopter transport units. Paramedics and registered nurses underwent a standardized training session including education on the guidelines for TXA candidate identification, the protocol for TXA administration, and the TXA known side-effect profile. Additionally, a system of access to real-time consultation with senior physicians familiar with study protocol at each participating trauma center was established prior to study initiation to address any first responder concerns regarding patient selection or TXA administration.

TXA was delivered in two doses following the protocol used in the CRASH-2 trial.^13^,^14^ The first dose was 1 gram of TXA in 100 ml of 0.9% normal saline infused over 10 minutes via intravenous (IV) or intraosseous access. This first dose was administered by paramedics or registered nurses as soon as feasible after patient assessment. Identification of study patients receiving TXA was achieved through a wristband labeled “TXA”, verbal communication at patient hand off by EMS, and/or by EMS run sheet. Following arrival to a participating trauma center, patients who received prehospital TXA were identified and re-assessed by trauma team members for signs of continued hemorrhagic shock. Patients who continued to meet the study criteria (Figure 1) received a second dose of 1 gram of TXA in 100 ml of 0.9% normal saline infused over eight hours via IV infusion. A patient may have received only one dose of TXA if they arrived to the trauma center and no longer met study criteria (Figure 1). We excluded from the study patients who were deceased upon arrival (declared dead on arrival with minimal resuscitation effort or failed to respond to resuscitation after 15 minutes in the ED), those who received TXA for non-trauma indications, and those who received TXA and were determined to be less than 18 years old upon arrival.

The control group was formed of patients seen at each receiving center within five years prior to the conclusion of data collection (June 2012 to July 2017). This group included patients who were not administered TXA because they were brought in by an EMS provider group not carrying TXA or because they were transported to the hospital by any means other than a designated EMS provider (e.g., friends, family, self). The control group patients met the same study criteria (Figure 1) as those in the TXA group. The control group patients were matched to TXA group patients through propensity scoring based upon gender, age, Injury Severity Score (ISS), and mechanism of injury. We further aimed to match TXA group patients with controls from the same trauma center.

The primary outcome was mortality measured at 24 hours, 48 hours, and 28 days. Additional variables included total blood products transfused during the hospital stay, the hospital and intensive care unit (ICU) length of stay (LOS), systolic blood pressure taken prior to TXA administration, Glasgow Coma Score observed prior to the first TXA dose in the field, and the incidence of known adverse events associated with TXA administration including thromboembolic events (e.g., deep vein thrombosis, pulmonary embolism), myocardial infarction, and neurological events (e.g., stroke, seizure).

Data for included subjects were abstracted from the electronic medical record and trauma registry for each patient. Follow up to determine mortality outcomes after hospital discharge was abstracted from the electronic medical record and trauma registry. In select cases, direct chart review was conducted, and in cases of missing data, study investigators contacted patients’ and/or patients’ families directly to determine survival outcomes. Estimated time to TXA administration by EMS was determined to be the estimated time of injury based on the time that the 911 call was received and documented time of TXA administration on the EMS run sheet.

### Statistical Analysis

We conducted all statistical analyses using the SAS software for Windows version 9.3 (SAS Institute, Cary, North Carolina, USA). Descriptive statistics were presented as means and standard deviations for continuous variables, along with frequencies and proportions for categorical variables. Propensity score matching based on age, gender, ISS, and mechanism of injury were used to form the TXA and control groups. Matching of each patient for the TXA group and control group was performed within the trauma registry of each center involved. We conducted chi-square analyses to identify whether there was a difference in the mortality at 24 hours, 48 hours, and 28 days between the TXA and control groups. Independent T-tests were conducted to identify whether there were differences of continuous variables (e.g., age) between the TXA and control groups.

Wilcoxon rank-sum tests were conducted to identify whether the median of some continuous variables (e.g., hospital LOS) was different between the TXA and control groups. Based on the original study design, we conducted three subgroup analyses to assess patient outcomes including (1) those who received one dose of TXA in comparison to two doses of TXA; (2) those who sustained significant blood loss (≥10 units of total blood products transfused) and those who did not sustain significant blood loss (<10 units of total blood products transfused), similar to the subanalysis performed in the Military Application of Tranexamic Acid in Trauma Emergency Resuscitation (MATTERs) study;^22^ (3) those who were severely injury (ISS ≥16) and those who were less severely injured (ISS <16).

The original sample-size calculation was based on the published results using 48-hour mortality as the primary outcome. Morrison and colleagues suggested that the TXA 48-hour mortality rates were 11.3% and 18.9% for TXA and control.^22^ Controlling for the type I error rate of 0.05, a sample size of 369 patients in each group would achieve a statistical power of 0.80.

## RESULTS

**Figure 2:**
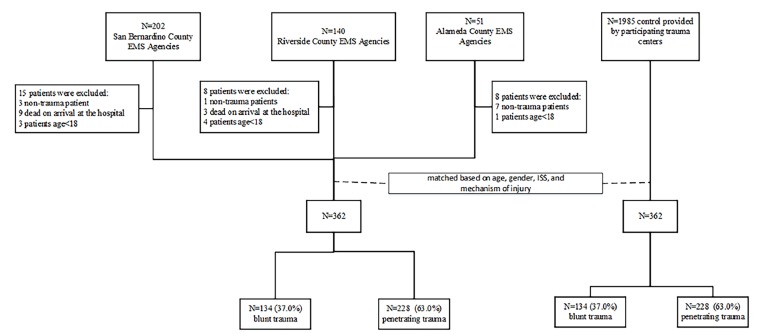
Patient flow chart.

A total of 362 patients were included in the TXA group (Figure 2). To eliminate the confounding effect of age, gender, ISS, and mechanism of injury, we conducted a propensity matching based on these four factors to select 362 patients as the control group. As a result, 724 patients were included in the final analysis. The median time for paramedics to administer TXA from the estimated time of injury was 33 minutes (interquartile range: 26 min, 46 min). As expected per the propensity matching process, there was no statistically significant difference in age (37.96 vs. 37.64 years for the TXA and control groups, respectively, difference=0.32 with 95% confidence interval [CI] [−2.05 to 2.69]), percentage of males (80.9% vs. 80.9% for the TXA and control groups, respectively, odds ratio [OR]=1 with 95% CI [0.69 to 1.45]), ISS (16.08 vs, 17.15 for the TXA and control groups, respectively, difference=−1.07 with 95% CI [−2.86 to 0.72]), and mechanism of injury (percentage of blunt trauma was 37.0% for both the TXA and control groups, respectively, OR=1 with 95% CI [0.74 to 1.35] (Table 1).

We compared clinical outcomes between the TXA and control groups. The results were also presented in Table 1. The TXA group had a statistically significant decrease in 28-day mortality (3.6% vs 8.3%, OR=0.41 with 95% CI [0.21 to 0.8]), fewer units of total blood products transfused (median of 1 vs. 3 units, difference=2 with 95% CI [1.14 to 2.86]), shorter hospital LOS (median of 4 vs. 8 days, difference=4 with 95% CI [2.35 to 5.64]), and shorter ICU length of stay (median of 4 vs. 5 days, difference=1 with 95% CI [0.65 to 2.25]).

Regarding the adverse events following TXA administration, no differences in the incidence of thromboembolic, myocardial infarction, or neurologic events were noted between the TXA and control groups. In the TXA group, two thromboembolic events, zero neurologic events, and zero myocardial infarction events were reported. In the control group, two thromboembolic events, zero neurologic events, and zero myocardial infarction events were reported. Additionally, two neurologic events were considered as possible adverse events in the TXA group, but after thorough review of each case, TXA as the primary etiology was deemed remote. In one case, a young male patient received TXA following a head-on, high-speed, motor vehicle accident where he sustained multiple, long bone fractures. He subsequently experienced a hemisphere ischemic stroke 40 hours after admission. Repeat computed tomography (CT) of his head revealed a new large ischemic infarct in the right middle cerebral artery distribution with moderate mass effect and midline shift. Suspecting traumatic vascular injury, a computed tomography angiography (CTA) study was ordered but not completed after his family decided to instate a do-not-resuscitate (DNR) order. A second case of ischemic stroke following TXA administration occurred in an elderly individual following a high-speed motor vehicle accident where the patient presented with altered mental status, scalp lacerations and a possible, small subdural hematoma as well as multiple, long bone fractures. Forty-eight hours after admission, the patient was diagnosed with an ischemic stroke, which neurosurgery attributed to fat emboli from long bone fractures.

We conducted a subgroup analysis to assess clinical outcomes between patients who received one dose vs. two doses of TXA (Table 2). Compared with patients who received one dose of TXA, those who received two doses of TXA required more blood transfusions (median of 0 vs. 3 units of blood product, difference=3 with 95% CI [1.34 to 4.67]).

A second subgroup analysis was conducted among patients who required transfusion (Table 3). Among patients who received <10 units of blood transfusion, the TXA group required fewer units of blood products transfused (median of 0 vs. 2 units, difference=2 with 95% CI [1.44 to 3.56]), had shorter hospital LOS (median of 4 vs. 8 days, difference=4 with 95% CI [2.28 to 5.73]), and shorter ICU LOS (median of 3 vs. 4 days, difference=1 with 95% CI [0.98 to 2.02]). Among patients who received ≥10 units of blood transfusion, the TXA group had a statistically significant decrease in mortality at 28 days (8.5% vs 23.2%, OR=0.31 with 95% CI [0.11 to 0.84]).

We conducted a third subgroup analysis based on patients’ ISS score (Table 4). Among patients with ISS <16, the TXA group had lower 24-hour mortality (0% vs. 2.6%, OR=0), fewer units of blood product transfused (median of 0 vs. 2.7 units, difference=2.7 with 95% CI [2.02 to 3.64]), shorter hospital LOS (median of 3 vs. 7 days, difference=4 with 95% CI [1.66 to 6.34]), and shorter ICU LOS (median of 3 vs. 5 days, difference=2 with 95% CI [0.59 to 3.41]). Among patients with ISS >16, the TXA group had statistically significant decrease in 28-day mortality (6% vs 14.5%, OR=0.37 with 95% CI [0.17 to 0.8]).

## DISCUSSION

This prospective, observational cohort study with a retrospective comparison investigated the use of prehospital TXA in cases of traumatic hemorrhagic shock and suggested that prehospital TXA use was associated with improved survival outcomes. Reduced mortality was observed at 28 days. To our knowledge, this is the first large-scale, civilian study to systematically examine prehospital TXA administration in trauma patients in North America.

The mortality reduction noted in this study may be attributed to the antifibrinolytic properties of TXA. Evidence suggests that up to 15% of trauma patients may be in a state of hyperfibrinolysis at the scene of injury as noted on rotational thromboelastometry (ROTEM) and more than half of trauma patients may be in a state of moderate to severe fibrinolysis upon arrival to the hospital.^5^,^7^–^9^,^11^,^23^ These coagulopathies often begin within minutes of injury and worsen during transportation from the scene to the hospital.^7^,^9^,^11^ This process can threaten clot integrity and result in increased blood loss, morbidity, and mortality.^8^,^9^ The antifibrinolytic properties of TXA may act to slow or stop progression of coagulopathies that contribute to excessive blood loss and disruption of hemodynamic stability.

The current study showed a reduction in the total blood products transfused in those administered TXA. However, TXA appears to exert an effect beyond 24 hours, after the risk of bleeding has decreased.^3^ This may be a result of the anti-inflammatory effects of TXA that are mediated through a reduction in the magnitude of the plasmin level, thus reducing the pro-inflammatory effect of plasmin.^24^,^25^ This may be responsible for the observed trend toward decreased mortality at 48 hours and longer. Though the exact mechanism is not clear, current evidence demonstrates that the therapeutic mechanism of TXA is likely multifactorial in nature.

In particular, severely injured trauma patients appear to benefit most from TXA. This may be attributed to an increased incidence of acute coagulopathies among patients who have sustained severe traumatic injury as detected on ROTEM.^7^,^9^,^26^ Thesuinger et al. showed significant deterioration of relevant ROTEM clot parameters between the scene and hospital when TXA was not administered.^7^ However, Kunze-Szikszay et al. conducted a follow up study by assessing for acute coagulopathies noted on ROTEM in severely injured trauma patients before and after prehospital TXA administration.^12^ Despite no ROTEM changes following prehospital TXA, Kunze-Szikszay et al. concluded that TXA might have reduced unnecessary fibrinogen consumption due to fibrinolysis after comparing their results to those of Theusinger et al. However, the study by Kunze-Szikszay et al. was limited by a small sample size.

Additionally, Moore et al. demonstrated that TXA use in severely injured patients might result in adverse outcomes in select patients in a state of fibrinolysis shutdown or hyperfibrinolysis.^8^ Nonetheless, multiple other investigations of TXA use in the civilian prehospital and hospital settings found that TXA was most beneficial among severely injured trauma patients.^19^,^20^,^27^ Though TXA use in severely injured trauma patients may be beneficial, it appears that both the exact candidate-selection criteria and mechanism of action conferring benefit remain unclear. In addition, mortality in this study may be biased due to differences in mechanism and complexity of injuries sustained by patients.

To date, CRASH-2 represents the only randomized controlled trial assessing TXA in civilian adult trauma.^13^ The CRASH-2 findings suggested that TXA administered in the hospital within three hours of injury led to a decrease in all-cause mortality by 1.5% at 28 days. The current study demonstrated a decrease in mortality of 4.7% at 28 days. The corresponding number needed to treat was 22. One major difference between the two studies was the location that TXA was given and the timing of administration. By giving TXA in the prehospital setting, this significantly reduced the time to first dose from 2.8 hours in CRASH-2 to 33 minutes. Further, lack of standardized inclusion protocols between hospitals, many of which were part of underdeveloped trauma systems, along with unclear reporting of adverse events and injury severity, may have impacted the CRASH-2 findings.^19^,^20^

In regard to assessing the known side-effect profile associated with TXA use, the majority of studies note a limited incidence of adverse events. Though controversial, the CRASH-2 trial reported no increase in thromboembolic events in hospital patients given TXA.^13^ Among other observational studies assessing prehospital TXA in the civilian setting, no increases in multiple organ failure, sepsis, or thromboembolic events were noted.^19^,^20^ Notably, a slight increase in thromboembolic events following TXA was noted in a retrospective study in the combat setting; however, authors postulated that a higher injury burden in this setting may have resulted in this finding.^11^ The current study showed no significant increase in adverse events following TXA administration.

Notably, two aforementioned neurologic events occurred in patients receiving TXA; however, direct causation between TXA use and each neurologic event was deemed remote, though it could not be definitely excluded. In the first case, a DNR order by the family prevented definitive imaging to assess for traumatic vascular injury vs. a thromboembolic complication secondary to TXA leading to an ischemic stroke. The latter was considered more likely with respect to timing at nearly 40 hours after TXA. Similar to the first case, the second case had a severe mechanism of injury as well as multiple, long bone fractures that likely led to an ischemic stroke that occurred 48 hours after hospital admission. With respect to the mechanism and timing of this neurologic event, direct association with TXA administration appeared to be a less likely etiology, although it cannot be completely excluded. Additionally, no increase in hospital or ICU LOS was noted in the current study, further supporting a relatively non-complicated course among patients administered TXA.

The exact dosing of TXA for traumatic injury remains unclear.^23^ A fixed 1 gram dose administered in the field followed by a possible maintenance dose was deemed most practical in the emergency setting.^13^ In the current study, 64.9% of patients were administered only the first dose of TXA. This may have occurred when a patient no longer satisfied the inclusion criteria for a second TXA dose upon arrival to a participating trauma center. No difference in mortality was observed between those receiving one dose vs. two doses of TXA. If sufficient antifibrinolytic and anti-inflammatory effects occur with only a single dose of TXA, this challenges the apparent need for a maintenance dose. With respect to drug half-life, the duration is unclear in present literature ranging from two to eight hours depending on the dosage.^28^–^30^

Lastly, our study did not employ coagulation testing before prehospital TXA administration to determine if patients were indeed in a state of hyperfibrinolysis. This significantly limited our ability to administer TXA in a selective fashion. Given the study design and current limitations of point-of-care thromboelastography (TEG) or ROTEM testing, it would have been infeasible to employ such testing in the prehospital setting. Further, previous studies noted the incidence of moderate to severe fibrinolysis at the scene and upon hospital arrival to be over 50%, with fibrinolysis steadily worsening from the scene to the hospital when measured on ROTEM.^7^–^9^ Theusinger et al. concluded that monitoring coagulation via ROTEM at the scene of a trauma would not provide any clinically significant information in the majority of trauma patients.^7^ However, upon arrival to the receiving center, growing (but weak) evidence exists suggesting that point-of-care TEG or ROTEM may guide in any additional TXA dosing and blood product administration in critically ill patients.^12^,^31^ At present, administering TXA empirically to those with signs of hemorrhagic shock may be an effective practice until more prehospital point-of-care diagnostic techniques are available.

## LIMITATIONS

First, this study was limited by design. The prospective, non-randomized, cohort design did not allow TXA to be administered in a blinded fashion. Prehospital providers and physicians were aware that TXA had been administered, which may have slightly affected the level of care provided. However, given that the primary outcome was mortality, this impact was likely minimal. Additionally, while we did examine the adverse effects of TXA administration and report our findings, the original study was not powered based on the side effects of TXA administration.

Second, this study relied upon prehospital providers’ ability to accurately recognize signs of trauma-related hemorrhagic shock in the field, even if active external bleeding was not present. Despite thorough didactic training and distribution of study protocols, high injury acuity and/or inexperience may have resulted in some providers improperly selecting TXA candidates. Incidences of improper exclusion during the initial months were estimated at <4%. Through active troubleshooting, real-time physician support, and additional education sessions, the estimated incidence was reduced to <2% at study conclusion.

Third, we acknowledge that we were not able to account for certain potential confounding factors. In the prehospital setting, we did not account for the impact of total EMS transport time, availability of IV access, first responder prehospital interventions, or differences in the transporting provider agency. With regard to transport times, shorter times may have impacted the ability of first responders to establish IV access and/or administer TXA prior to arriving to the trauma center. Differences in transporting provider agency may also have slightly impacted care due to differing of standard operating procedures; however, TXA protocols were uniform. We also acknowledge that multiple receiving trauma centers in different geographic area may have slightly impacted the patient care outcomes. We attempted to mitigate the influence of these factors by matching the majority of TXA group patients with control patients from the same center. Furthermore, there may have been minor differences in ICU LOS between the five-year, retrospective control group and current practice. However, there were no institutional changes in ICU policy that would have affected our outcomes. Without accounting for these factors, minimal inherent differences may exist between the TXA and control groups and limit the generalizability of these results.

## CONCLUSION

The current study noted reduced mortality at 28 days following the administration of prehospital TXA in patients with signs of traumatic hemorrhagic shock. We further noted a decrease in blood product transfused and shorter hospital and ICU LOS, without an increase in thromboembolic events. Finally, this study demonstrated that TXA can be effectively and feasibly administered by civilian prehospital providers and in accordance with North American emergency medicine standards. Our findings support the use of prehospital TXA in adult civilian traumatic injury with signs of hemorrhagic shock.

## Figures and Tables

**Table 1:** Patient outcomes for the control and TXA groups.

	TXA (n=362)	Control (n=362)	Statistic with 95% CI*
Mortality at 24 hours	7 (1.9%)	13 (3.6%)	0.53 (0.21, 1.34)
Mortality at 48 hours	10 (2.8%)	16 (4.4%)	0.61 (0.27, 1.37)
Mortality at 28 days	13 (3.6%)	30 (8.3%)	0.41 (0.21, 0.8)
Total blood products transfused (in units), median (Q1, Q3)	1 (0, 6)	3 (2, 8)	2 (1.14, 2.86)
Hospital LOS (in days), median (Q1, Q3)	4 (1, 12)	8 (5, 15)	4 (2.35,5.64)
ICU LOS (in days), median (Q1, Q3)	4 (2, 8)	5 (3, 8)	1(0.65, 2.25)
Adverse events			
Thromboembolic events	2	2	Not Applicable
Myocardial infarction events	0	0	Not Applicable
Neurologic events	0	0	Not Applicable
Penetrating trauma	228 (63%)	228 (63%)	1 (0.74,1.35)
Male	293 (80.9%)	293 (80.9%)	1 (0.69, 1.45)
Age, years, mean ± SD	37.96 ± 16.11	37.64 ± 16.33	0.32 (−2.05, 2.69)
ISS, mean ± SD	16.08 ± 10.69	17.15 ± 11.71	−1.07 (−2.86, 0.72)
SBP, mmHg, mean ± SD	78.42 ± 16.17	83.66 ± 14.13	−5.24 (−8.48, −2)
GCS, mean ± SD	12.78 ± 3.71	13 ± 3.4	−0.22 (−1.01, 0.57)

*TXA*, tranexamic acid; *LOS*, length of stay; *ICU*, intensive care unit; *ISS*, Injury Severity Score; *SD*, standard deviation; *SBP*, systolic blood pressure; *GCS*, Glasgow Coma Scale Score; *OR*, odds ratio; *CI*, confidence interval; *Q1*, 25th percentile; *Q3*, 75th percentile.

* Reported as odds ratio and the corresponding 95% confidence interval or difference in median or mean between TXA and control groups, depending on the variable type.

**Table 2:** Subgroup analysis of the TXA group.

	Pre-hospital 1 dose of TXA (n=235)	1 Pre-hospital + 1 hospital dose of TXA (n=127)	Statistic with 95% CI*
Mortality at 24 hours	5 (2.1%)	2 (1.6%)	1.36 (0.26, 7.1)
Mortality at 48 hours	8 (3.4%)	2 (1.6%)	2.2 (0.46, 10.53)
Mortality at 28 days	9 (3.8%)	4 (3.2%)	1.22 (0.37, 4.06)
Total blood products transfused (in units), median (Q1, Q3)	0 (0, 3)	3 (0, 13)	3 (1.34, 4.67)
Hospital LOS (in days), median (Q1, Q3)	4 (1, 10)	6 (2, 15)	2 (−0.57, 4.58)
ICU LOS (in days), median (Q1, Q3)	3 (2, 5)	4 (2, 12)	1 (−1.07, 3.07)
Penetrating trauma	151 (64.3%)	77 (60.6%)	1.17 (0.75,1.82)
Male	188 (80%)	105 (82.7%)	0.84 (0.48, 1.47)
Age, years, mean ± SD	37.45 ± 16.62	38.76 ± 15.25	−1.31 (−4.81, 2.19)
ISS, mean ± SD	15.69 ± 10.77	16.81 ± 10.53	−1.14 (−3.45, 1.18)
SBP, mmHg, mean ± SD	80.53 ± 16	74.96 ± 15.94	5.57 (1.49, 9.65)
GCS, mean ± SD	12.73 ± 3.81	12.87 ± 3.53	−0.14 (−0.97, 0.69)

*TXA*, tranexamic acid; *LOS*, length of stay; *ICU*, intensive care unit; *ISS*, Injury Severity Score; *SD*, standard deviation; *SBP*, systolic blood pressure; *GCS*, Glasgow Coma Scale Score; *OR*, odds ratio; *CI*, confidence interval; *Q1*, 25th percentile; *Q3*, 75th percentile.

* Reported as odds ratio and the corresponding 95% confidence interval or difference in median or mean between TXA and control groups, depending on the variable type.

**Table 3:** Subgroup analysis of patients based on the number of units of blood product transfused.

	<10 units of blood transfused (n=584)			≥10 units of blood transfused (n=140)		
	
	TXA (n=291)	Control (n=293)	Statistic with 95% CI*	TXA (n=71)	Control (n=69)	Statistic with 95% CI*
Mortality at 24 hours	3 (1.0%)	7 (2.4%)	0.43 (0.11, 1.66)	4 (5.6%)	6 (8.7%)	0.63 (0.17, 2.33)
Mortality at 48 hours	5 (1.7%)	7 (2.4%)	0.72 (0.22, 2.28)	5 (7%)	9 (13%)	0.51 (0.16, 1.59)
Mortality at 28 days	7 (2.4%)	14 (4.8%)	0.49 (0.2, 1.24)	6 (8.5%)	16 (23.2%)	0.31 (0.11, 0.84)
Total blood products transfused (in units), median (Q1, Q3)	0 (0, 2)	2 (2, 4.3)	2 (1.44, 3.56)	18 (14, 32)	20 (14, 31)	2 (−2.76, 2.76)
Hospital LOS (in days), Median (Q1, Q3)	4 (1, 8)	8 (5, 15)	4 (2.28, 5.73)	13 (5, 22)	10 (6, 14)	3 (−2.76, 2.76)
ICU LOS (in days), median (Q1, Q3)	3 (2, 5.5)	4 (3, 8)	1 (0.98, 2.02)	5 (3, 14)	6 (4, 8)	1 (−1.87, 5.86)
Penetrating trauma	192 (66.0%)	175 (59.7%)	1.31 (0.93,1.83)	36 (50.7%)	53 (76.8%)	0.31 (0.15, 0.64)
Male	236 (81.1%)	230 (78.5%)	1.18 (0.78,1.76)	57 (80.3%)	63 (91.3%)	0.39 (0.14,1.08)
Age, years, mean ± SD	37.99 ± 16.3	38.26 ± 16.65	−0.27 (−3.01, 2.47)	37.87 ± 15.49	35 ± 14.68	2.87 (−1.85,7.59)
ISS, mean ± SD	14.77 ± 10.34	15.66 ± 10.28	−0.89 (−2.86, 1.08)	21.39 ± 10.51	24.81 ± 13.96	−3.42 (−7.4, 0.57)
SBP, mmHg, mean ± SD	79.61 ± 16.12	84.69 ± 14.17	−5.08 (−8.64, −1.51)	72.73 ± 15.36	78.88 ± 13.19	−6.15 (−13.57, 1.27)
GCS, mean ± SD	13.16 ± 3.42	13.25 ± 3.09	−0.09 (−0.91, 0.73)	11.21 ± 4.44	11.95 ± 4.39	−0.74 (−2.94, 1.46,)

*TXA*, tranexamic acid; *LOS*, length of stay; *ICU*, intensive care unit; *ISS*, Injury Severity Score; *SD*, standard deviation; *SBP*, systolic blood pressure; *GCS*, Glasgow Coma Scale Score; *OR*, odds ratio; *CI*, confidence interval; *Q1*, 25th percentile; *Q3*, 75th percentile.

* Reported as odds ratio and the corresponding 95% confidence interval or difference in median or mean between TXA and control groups, depending on the variable type.

**Table 4:** Subgroup analysis of patients based on the Injury Severity Score.

	Patients with ISS <16 (n=384)			Patients with ISS ≥16 (n=340)		
	
	TXA (n=194)	Control (n=190)	Statistic with 95% CI*	TXA (n=168)	Control (n=172)	Statistic with 95% CI*
Mortality at 24 hours	0 (0%)	5 (2.6%)	0	7 (4.2%)	8 (4.7%)	0.89 (0.32, 2.52)
Mortality at 48 hours	1 (0.5%)	5 (2.6%)	0.19 (0.02, 1.66)	9 (5.4%)	11 (6.4%)	0.83 (0.37, 2.05)
Mortality at 28 days	3 (1.6%)	5 (2.6%)	0.58 (0.14, 2.47)	10 (6%)	25 (14.5%)	0.37 (0.17, 0.8)
Total blood products transfused (in units), median (Q1, Q3)	0 (0, 2)	2.7 (2, 6)	2.7 (2.02, 3.64)	4 (0, 15)	4 (2, 12)	0 (−1.89, 1.89)
Hospital LOS (in days), median (Q1, Q3)	3 (1, 6)	7 (4, 13)	4 (1.66, 6.34)	8 (2, 16)	10 (6, 17)	2 (−0.89, 4.89)
ICU LOS (in days), median (Q1, Q3)	3 (2, 5)	5 (3, 9.5)	2 (0.59, 3.41)	5 (2, 13)	5 (3, 8)	0 (−2.22, 2.22)
Penetrating trauma	140 (72.2%)	132 (70.0%)	1.14 (0.73,1.77)	88 (52.4%)	96 (55.8%)	0.87 (0.57, 1.33)
Male	157 (80.9%)	152 (80%)	1.06 (0.64,1.76)	136 (81%)	141 (82%)	0.93 (0.54, 1.62)
Age, years, mean ± SD	38.67 ± 16.68	38.95 ± 17.41	−0.28 (−4.06, 3.5)	36.72 ± 15.42	36.97 ± 15.07	−0.25 (−3.36, 2.86)
ISS, mean ± SD	8.61 ± 2.91	9.27 ± 2.89	−0.66 (−1.33, 0.01)	26.28 ± 9.97	26.65 ± 11.73	−0.37 (−2.72, 1.98)
SBP, mmHg, mean ± SD	78.7 ± 16.12	87.3 ± 19.09	−8.6 (−16.44, −0.76)	78.11 ± 16.29	83.77 ± 12.44	−5.66 (−9.41, −1.92)
GCS, mean ± SD	13.27 ± 3.21	14.72 ± 4.24	−1.45 (−2.96, 0.06)	12.22 ± 4.15	12.77 ± 3.53	−0.45 (−1.49, 0.58)

*TXA*, tranexamic acid; *LOS*, length of stay; *ICU*, intensive care unit; *ISS*, Injury Severity Score; *SD*, standard deviation; *SBP*, systolic blood pressure; *GCS*, Glasgow Coma Scale Score; *OR*, odds ratio; *CI*, confidence interval; *Q1*, 25th percentile; *Q3*, 75th percentile.

* Reported as odds ratio and the corresponding 95% confidence interval or difference in median or mean between TXA and control groups, depending on the variable type.
